# Successes and limitations of pretrained YOLO detectors applied to unseen time-lapse images for automated pollinator monitoring

**DOI:** 10.1038/s41598-025-16140-z

**Published:** 2025-08-21

**Authors:** Valentin Ștefan, Thomas Stark, Michael Wurm, Hannes Taubenböck, Tiffany M. Knight

**Affiliations:** 1 Department of Species Interaction Ecology, Helmholtz Centre for Environmental Research - UFZ, Leipzig, Germany; 2 German Centre for Integrative Biodiversity Research (iDiv) Halle-Jena-Leipzig, Leipzig, Germany; 3 Institute of Biology, Martin Luther University Halle-Wittenberg, Halle (Saale), Germany; 4 German Remote Sensing Data Center (DFD), German Aerospace Center (DLR), Oberpfaffenhofen, Germany; 5 Department of Global Urbanisation and Remote Sensing, University of Würzburg, Würzburg, Germany; 6 Department of Science and Conservation, National Tropical Botanical Garden, Kalāheo, USA

**Keywords:** Pollinator detection, Automated insect monitoring, Out-of-distribution generalisation, YOLO detectors, Smartphone images, Time-lapse images, Ecological networks, Machine learning, Ecology

## Abstract

Pollinating insects provide essential ecosystem services, and using time-lapse photography to automate their observation could improve monitoring efficiency. Computer vision models, trained on clear citizen science photos, can detect insects in similar images with high accuracy, but their performance in images taken using time-lapse photography is unknown. We evaluated the generalisation of three lightweight YOLO detectors (YOLOv5-nano, YOLOv5-small, YOLOv7-tiny), previously trained on citizen science images, for detecting ~ 1,300 flower-visiting arthropod individuals in nearly 24,000 time-lapse images captured with a fixed smartphone setup. These field images featured unseen backgrounds and smaller arthropods than the training data. YOLOv5-small, the model with the highest number of trainable parameters, performed best, localising 91.21% of Hymenoptera and 80.69% of Diptera individuals. However, classification recall was lower (80.45% and 66.90%, respectively), partly due to Syrphidae mimicking Hymenoptera and the challenge of detecting smaller, blurrier flower visitors. This study reveals both the potential and limitations of such models for real-world automated monitoring, suggesting they work well for larger and sharply visible pollinators but need improvement for smaller, less sharp cases.

**Supplementary Information:**

The online version contains supplementary material available at 10.1038/s41598-025-16140-z.

## Introduction

 Pollinators play a crucial role in sustaining our ecosystems and ensuring food security. Yet they face an alarming decline^1,2^which has the potential to alter the structure of plant-pollinator interactions and the services that these pollinators provide^3^. Hence, there is a growing focus on understanding trends in pollinator abundance and diversity, along with plant-pollinator interaction structures, in order to comprehend the drivers of change and guide management strategies (e.g., the EU Pollinators Initiative^4^. Detecting trends requires standardised monitoring efforts over time and space. Traditional methods involve capturing pollinators and identifying them using microscopy^5,6^ or DNA barcoding^7^. However, these methods are resource-intensive and require killing the pollinators. In this context, emerging technologies in machine learning, computer vision and portable microcomputers have the potential to automate the monitoring of pollination^8^ and to do so in a non-lethal way^9^.

Recent advancements in computer vision, particularly in deep convolutional neural networks (CNNs), have seen a surge in popularity. A notable aspect of this trend is the considerable effort developers have invested in documenting the use of such architectures, exemplified by code bases like *Ultralytics*^10^*Detectron2*^11^ or *Pytorch-Wildlife*^12^. This, coupled with ongoing improvements in sensors, camera traps, smartphones and programmable microcomputers equipped with graphics processing units (GPUs, e.g., *Raspberry Pi 5*^13^, *Luxonis OAK modules*^14^*NVIDIA Jetson Nano Developer Kit*^15^*Coral Dev Board*^16^*Qualcomm Snapdragon*^17^, has expanded the application of CNNs in wildlife monitoring^18,19^. These technologies are also increasingly being utilised in pollination monitoring^8,20–25^.

CNN performance scales logarithmically with training dataset size^26^. However, these models typically show optimal generalisation primarily with data from imaging techniques similar to those used in training^27,28^. Their performance often drops when training and test data distributions differ^29–31^. This is less problematic if CNNs are applied to images closely resembling training data. For monitoring plant-pollinator interactions, cameras must be mounted above diverse flowers, inflorescences, or flower patches in varying field conditions. This presents a unique distribution shift challenge for CNNs trained for pollinator localisation and classification using images captured by citizen scientists^32^. Particularly, images from citizen-science platforms can exhibit bias, typically being well-lit and well-focused, with the subject usually centred and tightly framed^27,33^ as contributors are encouraged to upload their best images, and to crop around the target organism to aid community identification^34^. While these images can be used for training classifiers, they may pose challenges for developing generalisable object detectors that can be used for autonomous cameras mounted above flowers in field conditions, which will capture relatively small pollinators against complex floral backgrounds and with little to no user intervention.

CNN studies typically split an available image dataset into training, validation, and test sets, all sampled from the same distribution of images. In-distribution testing evaluates model performance on a test set drawn from this distribution. In contrast, Out-of-Distribution (OOD) testing evaluates models on unseen images from the same domain (e.g., pollinator monitoring) but with a shifted distribution^28,35,36^. While model performance is often assessed using an in-distribution test set, OOD tests better reveal a model’s ability to adapt to a wider range of images, providing a tougher, more realistic measure of its learning and generalisation skills.

For pollinating insects, images from citizen science platforms are an abundant source for training CNN models. We have shown, using an in-distribution test, that these models perform well in localising and classifying arthropods into broad groups, such as taxonomic orders^32^. We have also shown that a fixed setup using affordable smartphones, mounted on tripods above flowers and set to take time-lapse photos, can capture images of enough quality for experts to identify pollinators to these same broad groups and sometimes even to finer taxonomic levels^37^ (family, genus, and species). However, it remains unknown how well CNNs trained on citizen science images will perform at localising and classifying pollinating insects in field images taken with a fixed smartphone setup.

In this study, we evaluated the OOD generalisation capabilities of lightweight YOLO models (YOLOv5-nano, YOLOv5-small, and YOLOv7-tiny), trained and tested on curated citizen science images of flower-visiting arthropods^32^which are typically well focused, cropped and centred on the target organisms. Our OOD dataset consists of arthropod flower visitors interacting with the target flowers (which we refer to as pollinators even though flower visitors might not always perform pollination^38^. This focus on visitors that might perform pollination is in line with our aim to contribute to advancing pollinator monitoring. Generally, we assessed the efficacy of these models in localising and classifying pollinators captured in time-lapse sequences, comprising nearly 24,000 field images captured with a fixed smartphone setup. This OOD test set, where relatively smaller arthropods appear against unseen, complex floral backgrounds, presents a distribution shift from the training set.

Specifically, we first evaluated the three models for class-agnostic arthropod localisation across all images captured with the fixed smartphone setup. The best-performing model, selected based on F1 score, was then analysed further. Given the rarity of flower visitors in time-lapse images (an average of 6 pollinators per hour across our dataset), we tested the model’s false positive rate on a sample of floral-only background frames. Expecting arthropod bounding box area and image sharpness to affect performance, we compared their distributions between successful and unsuccessful localisation and classification outcomes. We assessed the best model’s ability to localise and classify individual pollinators across time-lapse sequences, a more relevant setting for pollination monitoring than independent frames. Diptera and Hymenoptera pollinators were the most common visitors in the dataset. We therefore assessed the model’s ability to distinguish between three groups of flower visitors: Diptera, Hymenoptera, and OtherT (other taxa). As hoverflies (Syrphidae, Diptera) mimic bees and wasps (Hymenoptera), we tested whether misclassifications between these two orders were more common than those with other groups. Such mimicry can cause high-confidence mislabels, where the model confidently but incorrectly assigns the pollinator within the bounding box to the wrong group. In contrast, smaller or blurrier pollinators tend to lower model confidence. To investigate these dynamics, we compared the model’s confidence, bounding box size, and image sharpness between correctly and incorrectly classified cases, focusing on Hymenoptera and Diptera taxa most frequently misclassified as each other.

## Methods

### Dataset

Time-lapse images of flower-visiting arthropods were automatically captured using smartphones from July to September 2021 in urban green spaces in and around Leipzig and Halle, Germany. The detailed methodology of data collection is provided in Ștefan et al.^37^. For these observations, smartphones were positioned above selected open flowers of 33 plant species. The smartphones captured time-lapse images at an average rate of approximately one frame every 1.6 ± 0.4 s (mean ± s.d) for an average session duration of approximately one hour (3,553 ± 372 s, mean ± s.d.) on a targeted flower^37^after which the smartphones were relocated to different flowers.

**Fig. 1:**
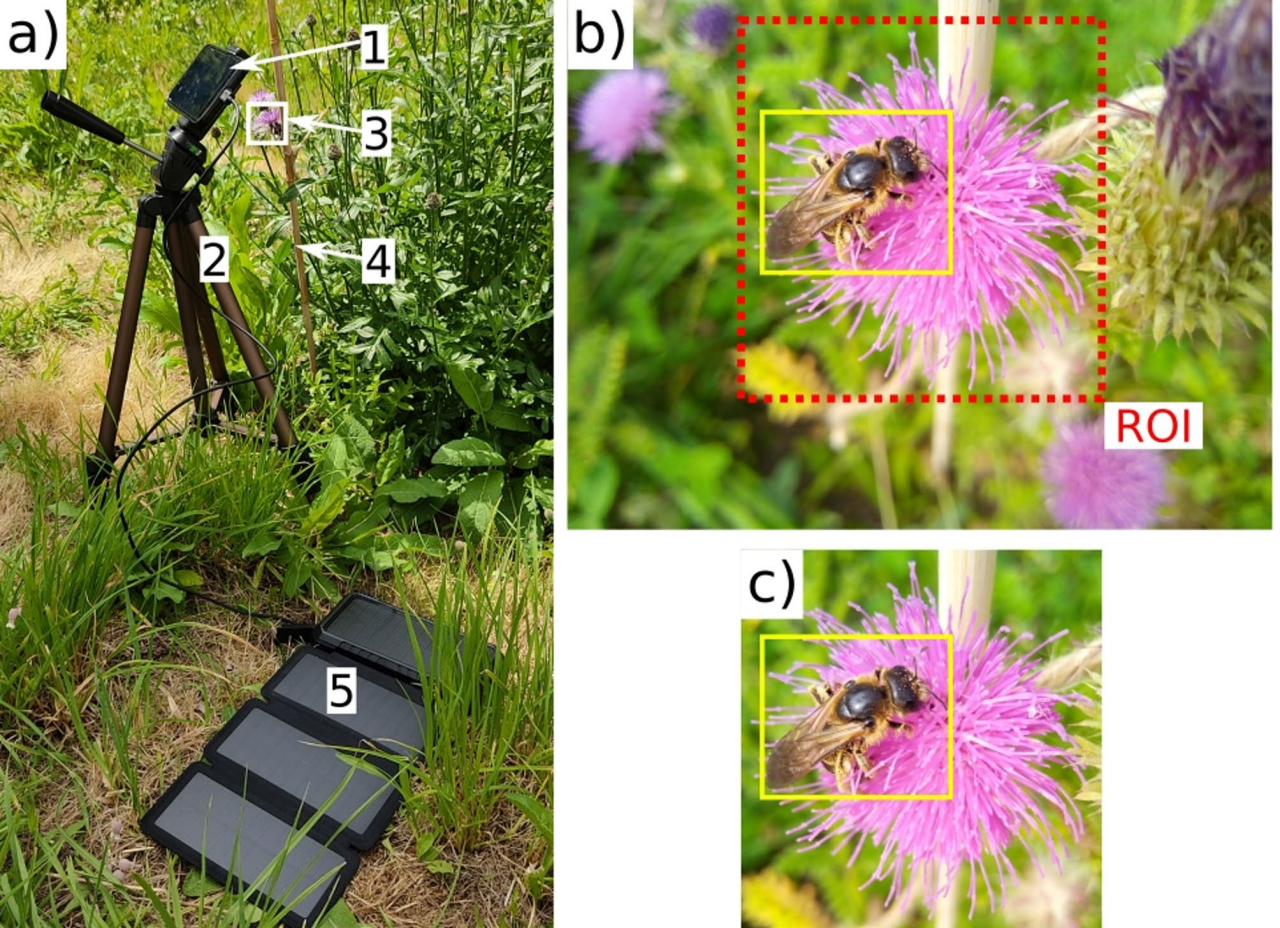
(**a**) Setup for time-lapse image capture featuring a smartphone (1) on a tripod (2) above the target flower (3), supported by a stick (4) to reduce wind motion and connected to a power bank (5) for continuous operation. (**b**) An original, full-frame image from the smartphone showing a pollinator and the target flower. (c) A cropped image highlighting the region of interest (ROI) used for analysis.

For stable mounting, smartphones were secured on tripods and continuously powered through USB cables connected to power banks (e.g., Fig. 1a).We used the OpenCamera app^39^ for time-lapse image capture. To ensure that the phone’s autofocus does not target the background instead of the flower, each recording session started with the focus fixed on the target flower and remained unadjusted until the end of the session. Furthermore, to mitigate wind-induced movements, flowers were anchored to wooden sticks with yarn. Smartphones were set 15–20 cm away from the centre of the target flower. Image acquisition was primarily at a resolution of 1600 × 1200 pixels (over 94% of images), with automatic exposure adjustment adapting to changing lighting conditions.

We visually parsed 213 distinct time-lapse sessions, each set against a unique floral background drawn from a selection of 33 different plant species, amassing a total of 460,056 time-lapsed images (see appendices in Ștefan et al.^37^. Subsequently, manual inspection of each image determined arthropod presence. When detected, a bounding box was drawn around the arthropod, and its taxonomic order was typed in using the VGG Image Annotator (VIA) software^40^. Because our focus was on monitoring pollinators on target flowers, a bounding box was placed around the target flower in each image containing an annotated arthropod, specifying the region of interest (ROI, Fig. 1b). In total, 33,502 (7.28%) images contained at least one arthropod, which resulted in 35,192 annotated arthropod bounding boxes. Of the images analysed, 94.85% contained only a single arthropod bounding box, and a maximum of four bounding boxes were found in a single image.

We excluded any bounding boxes annotated with the Thysanoptera order (thrips), as well as 11 boxes for which the arthropod order could not be identified. While thrips can be pollinators^41^the individuals in our dataset were typically very small (around 1 mm or less) and slender. Given their minute size relative to our camera’s field of view, these organisms were considered unlikely to be reliably localised and classified by a CNN in our field settings.

To focus on the ROI (i.e., the target flower), the original full-frame images were cropped (e.g., Fig. 1b, c). This cropping was guided by the union of the bounding boxes for both the ROI and the visiting arthropod, ensuring that target arthropods at the edges of the ROI were not cut off. Following this cropping and filtering process, the refined OOD dataset comprised 201 time-lapse sessions on top of flowers from 32 plant species, 23,899 images, and 24,656 arthropod bounding boxes (Table 1). It should be noted that 182 of these bounding boxes contained co-occurring arthropods that, while within or intersecting the ROI, did not interact with the target flower and were removed from the model evaluation. The final cropped images had an average size of 851 pixels in width and 796 pixels in height, and the original average dimensions were 1571 pixels wide and 1252 pixels high. The floral backgrounds in these images exhibited a long-tailed distribution, with 60.10% of arthropod bounding boxes (instances) located on flowers of just four plant species: *Centaurea jacea* (26.62%), *Daucus carota* (19.05%), *Clematis vitalba* (8.29%), and *Carduus acanthoides* (6.14%).

**Fig. 2:**
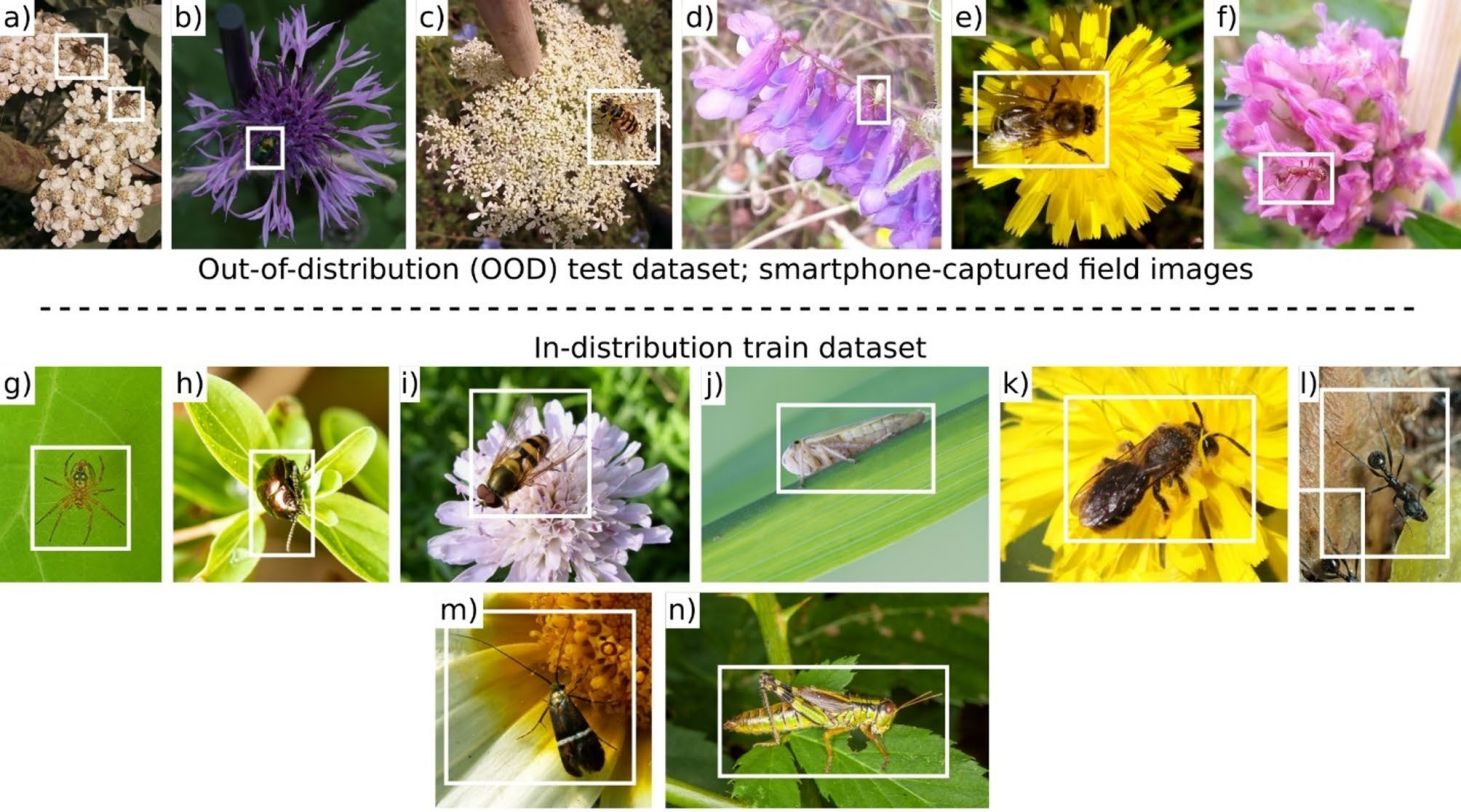
Example of cropped smartphone-captured images representing the six groups of flower visitors in our out-of-distribution (OOD) test dataset (a to f) vs. the in-distribution training dataset used in Stark et al.^32^ (g to n). Taxonomic orders in OOD test and train datasets: Arenae (a, g^42^, Coleoptera (b, h^43^, Diptera (c, i^44^, Hemiptera (d, j^45^, Hymenoptera (e, k^46^, Hymenoptera-Formicidae (f, l^47^. Lepidoptera (m^48^ and Orthoptera (n^49^ are exclusive to the training dataset. The average bounding box area in the OOD test set is approximately 4.5 times less than in the training dataset. The image backgrounds in the training dataset are more diverse, whereas the OOD test dataset features exclusively floral backgrounds.

A total of 1,281 unique arthropod individuals (each annotated as a series of bounding boxes across a time-lapse sequence of images) were identified in the OOD dataset, spanning six taxonomic groups: Hymenoptera (bees and wasps), Diptera (true flies), Coleoptera (beetles), Hymenoptera-Formicidae (ants), Araneae (spiders), and Hemiptera (true bugs), as detailed in Table 1 and shown in Fig. 2. Pollinators from Hymenoptera (except ants) and Diptera orders were identified to the lowest taxonomic level possible during a previous study^37^. Given the time-lapse methodology of our image collection, an individual arthropod might be present in a solitary image or persist across multiple images (e.g., Fig. 5). In our OOD dataset, instances ranged from a single bounding box to a case where an individual arthropod remained on a flower long enough to be captured in 1,710 time-lapse images, thus resulting in a series of 1,710 bounding boxes. The median number of bounding boxes per arthropod individual was seven, indicating a typical visit duration of approximately 11.2 s captured in our dataset. Each arthropod visible across consecutive time-lapse frames received a unique identifier, and small individuals traversing a target flower’s complex structure, if temporarily occluded by flower parts, retained the same identifier upon reappearance.

While the training dataset had an average relative bounding box area of 0.337, the average in the OOD test set is 4.5 times smaller, at 0.075. Furthermore, the disparity in medians is more pronounced with the median for the OOD dataset at 0.028, which is over ten times smaller than that of the training dataset at 0.288.

**Table 1:** Summary statistics for 1,281 arthropods in the OOD test set. The table enumerates counts of bounding Boxes (N. Box), their mean relative bounding Box area (Mean rel. Box area, proportions), counts and percentages of images (N. Img., N. Img. %), and individual arthropods (N. Ids.), alongside their respective percentages (N. Ids. %) and the cumulative Sum of these percentages (Cumul. Sum %). Note that the Sum of N. Img. Exceeds the total number of images in the OOD dataset due to the presence of multiple individuals from different categories in some images.

Pollination	Arthropod category	*N*. box	Mean rel. box area	*N*. img.	*N*. img. %	*N*. ids.	*N*. ids. %	Cumul. sum %
Common pollinators	Hymenoptera	13,254	0.107	13,084	54.75	1,013	79.08	79.08
	Diptera	5,018	0.071	4,998	20.91	145	11.32	90.40
Other flower visitors (OtherT); usually not pollinating	Coleoptera	2,778	0.010	2,770	11.59	20	1.56	91.96
	Formicidae	1,967	0.013	1,962	8.21	82	6.41	98.36
	Araneae	1,036	0.014	994	4.16	10	0.78	99.14
	Hemiptera	603	0.011	603	2.52	11	0.86	100
	Total	24,656		23,899	100	1,281	100	

### Model evaluation

In our previous work^32^we trained three YOLO object detection models, YOLOv5n (nano), YOLOv5s (small), and YOLOv7t (tiny), on a dataset of arthropod images primarily sourced from citizen science platforms, where photographers prioritise high-quality, carefully framed, detailed images (sometimes using telephoto lenses, favouring close-ups shots to ensure clear community identification^34^. These models were evaluated using a traditional data split approach, where the test images were in-distribution, meaning they originated from the same source as the training images and shared similar characteristics. In contrast, the current study evaluates these pre-trained models on a novel OOD dataset, with time-lapse images captured passively using a fixed smartphone setup, without real-time human selection, modified only by cropping to the ROI.

As a first step, we selected the model with the highest F1 score (harmonic mean of precision and recall) for the task of arthropod localization in single images, treating all predictions as a single “arthropod” class, irrespective of their time-lapse sequence. Model selection involved a grid search across non-maximum suppression (NMS) intersection-over-union (IoU) from 0.1 to 0.9 in increments of 0.1. For each configuration we computed precision, recall, F1 scores across prediction confidence thresholds (F1-confidence curves), and the area under the precision-recall curve (AUC). Further implementation details, including the definitions of True Positives (box-TP), False Positives (box-FP), and False Negatives (box-FN), are presented in the Supplementary Methods and Supplementary Fig. S2. Additionally, non-maximum suppression (NMS) specifics are further elaborated in Supplementary Fig. S1. Inference on the OOD dataset was conducted at an image size of 640 × 640 pixels, consistent with the training image dimensions from our previous study^32^.

Subsequently, we employed the optimised detector with the highest F1 score for inference on the OOD dataset, now evaluating predictions across all classes. At this step, we assessed the model’s ability to both localise and classify the 1,281 individual arthropods. In this context, an individual arthropod was defined as a series of bounding boxes marked in successive images throughout the time-lapse sequence, which captured the arthropod’s presence across multiple frames (e.g., Fig. 5). Consequently, in these cases, we will refer to the process as *individual arthropod localisation* or *classification* in subsequent discussions. Conversely, when discussing *arthropod box localisation* or *classification*, we are referring specifically to the best model’s ability to localise or classify an arthropod instance within a given image, regardless of the time-lapse sequence (that is, consecutive time-lapse images are considered *independent* from each other).

The possible prediction labels for arthropod classification given by the pre-trained YOLO weights^32^ were Araneae (spiders), Coleoptera (beetles), Diptera (true flies), Hemiptera (true bugs), Hymenoptera (bees and wasps), Hymenoptera Formicidae (ants), Lepidoptera (moths and butterflies), and Orthoptera (crickets and grasshoppers). Despite being potential prediction labels, Lepidoptera and Orthoptera do not appear in the OOD dataset. For analysis at the individual arthropod level, we used three groups: Hymenoptera, Diptera, and OtherT, comprising the remaining taxa groups.

Successful localisation of an individual arthropod across sequences (arthropod-TP) was achieved if at least one box-TP was encountered across the time-lapse sequence, regardless of the predicted labels (e.g., Fig. 5), indicating a successful floral visit.

To evaluate the individual arthropod classification performance of the best detector, we employed a maximum confidence rule for label assignment across an entire time-lapse sequence. Specifically, when multiple predicted box-TPs across the sequence correspond to the same arthropod, the label with the highest YOLO confidence score was selected. Subsequently, performance metrics including precision, recall, F1-score and accuracy were computed for each arthropod category and overall, weighted by the number of individuals.

Additionally, we employed the best detector to assess false positives per image (FPPI) on the OOD images that only contained floral backgrounds. This detection test utilised 212 background images selected from the 213 distinct time-lapse sessions, with one session excluded because all images contained a beetle. FPPI was then defined as the total number of FPs divided by the total number of images in the test set.

We applied a one-tailed exact binomial test using the “binom.test()” function in R^50^ to assess whether cross-order Hymenoptera-Diptera misclassifications occurred at a frequency significantly higher than expected by chance, specifically testing for an excess over chance levels. For the independent frames analysis, where the YOLO model classified arthropod instances into eight groups (Araneae, Coleoptera, Diptera, Hemiptera, Hymenoptera, Hymenoptera-Formicidae, Lepidoptera, Orthoptera), each class had seven possible misclassifications, giving an expected probability of 1/7, 14.29%. For the individual arthropod analysis, where arthropods were observed across frames and grouped into Hymenoptera, Diptera, and OtherT, misclassifications had two possible outcomes (expected probability: 1/2, 50%). The test determined whether these misclassifications occurred significantly more often than expected (*p* < 0.05).

To quantify image sharpness within bounding boxes, we applied the Sobel-Tenengrad operator as a proxy^51^implementing it using the “cv2.Sobel()” function from the OpenCV library^52^ within Python 3^53^. Higher values indicate more edges, signifying increased sharpness. Due to the large absolute values, we normalised them to a 0–1 range (blur to sharp) by dividing each by the maximum observed value.

To assess differences in relative bounding box area and normalised image sharpness for localisation and classification tasks, we implemented a nonparametric permutation test. The custom R code for this analysis is available on our GitHub repository. This test examines whether the means and medians of two distributions differ, assuming under the null hypothesis that the distributions are identical, with expected differences in these metrics being zero. We compared two groups: (1) ground truth arthropod boxes that were either localised or not, and (2) among localised instances, those correctly classified versus misclassified. We selected this test due to the long-tailed distributions, which deviate from normality. For each comparison, we reported the observed difference (Δ, absolute value) and the p-value relative to the 0.05 significance threshold. The p-value was computed as the proportion of permuted differences at least as extreme as the observed difference, with 1,000 permutations.

## Results

**Fig. 3:**
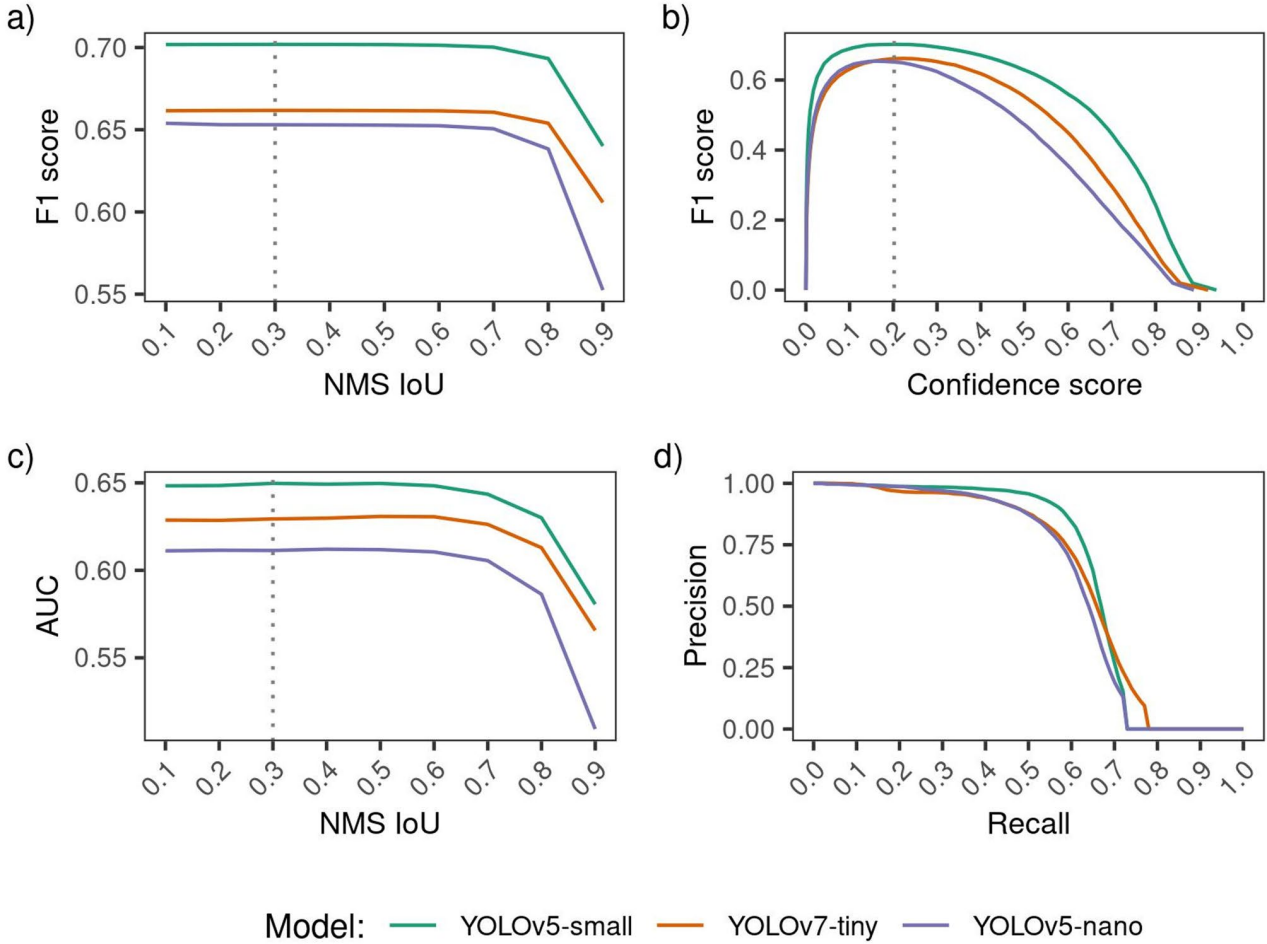
Grid search results for the optimal NMS confidence and NMS-IoU hyperparameters for YOLO detectors (localisation task, independent frames), with a focus on the maximum F1 score (panels a and b) and area under the precision-recall curve (AUC, panels c and d). The YOLOv5-small model demonstrates superior performance (highest F1 and AUC), achieving optimal detection at an NMS confidence estimate of 0.2019 (panel b) and a NMS-IoU of 0.3 (panels a and c), marked with grey dotted vertical lines. The presented F1-confidence curve (panel b) and the precision-recall curve (panel d) correspond to the optimal NMS-IoU for each model. The evaluation was performed using an eval-IoU of 0.5.

In the initial class-agnostic test assessing arthropod box localisation within independent frames, YOLOv5-small outperformed YOLOv7-tiny and YOLOv5-nano (Fig. 3, Supplementary Table S1). Grid search optimisation of YOLOv5-small estimated a maximum F1 score of 0.7019 and an AUC of 0.6497 at optimal NMS hyperparameters IoU = 0.3 (Fig. 3a, c) and confidence score = 0.2019 (Fig. 3b). This F1 optimisation also maximised AUC (Fig. 3c, d). Performance remained stable until NMS-IoU exceeded 0.6, then declined (Fig. 3a, c). In our prior study^32^ with citizen science test images (similar to the training set), optimal NMS-IoU was 0.6 and confidence was 0.3. There, YOLOv5-small achieved a higher F1 score of 0.8886, followed by YOLOv7-tiny (0.8672) and YOLOv5-nano (0.8366), mirroring the current ranking.

At the standard evaluation IoU (eval-IoU) threshold of 0.5, the model produced 2,265 false positive boxes (box-FPs) across the 23,899 OOD arthropod images, yielding a FPPI of 9.48%. At eval-IoU 0.1, box-FPs decreased to 1,799 (FPPI = 7.53%). In the control test on 212 floral background images (without arthropods), the model generated 16 box-FPs (FPPI = 7.55%), each occurring in a separate image.

**Fig. 4:**
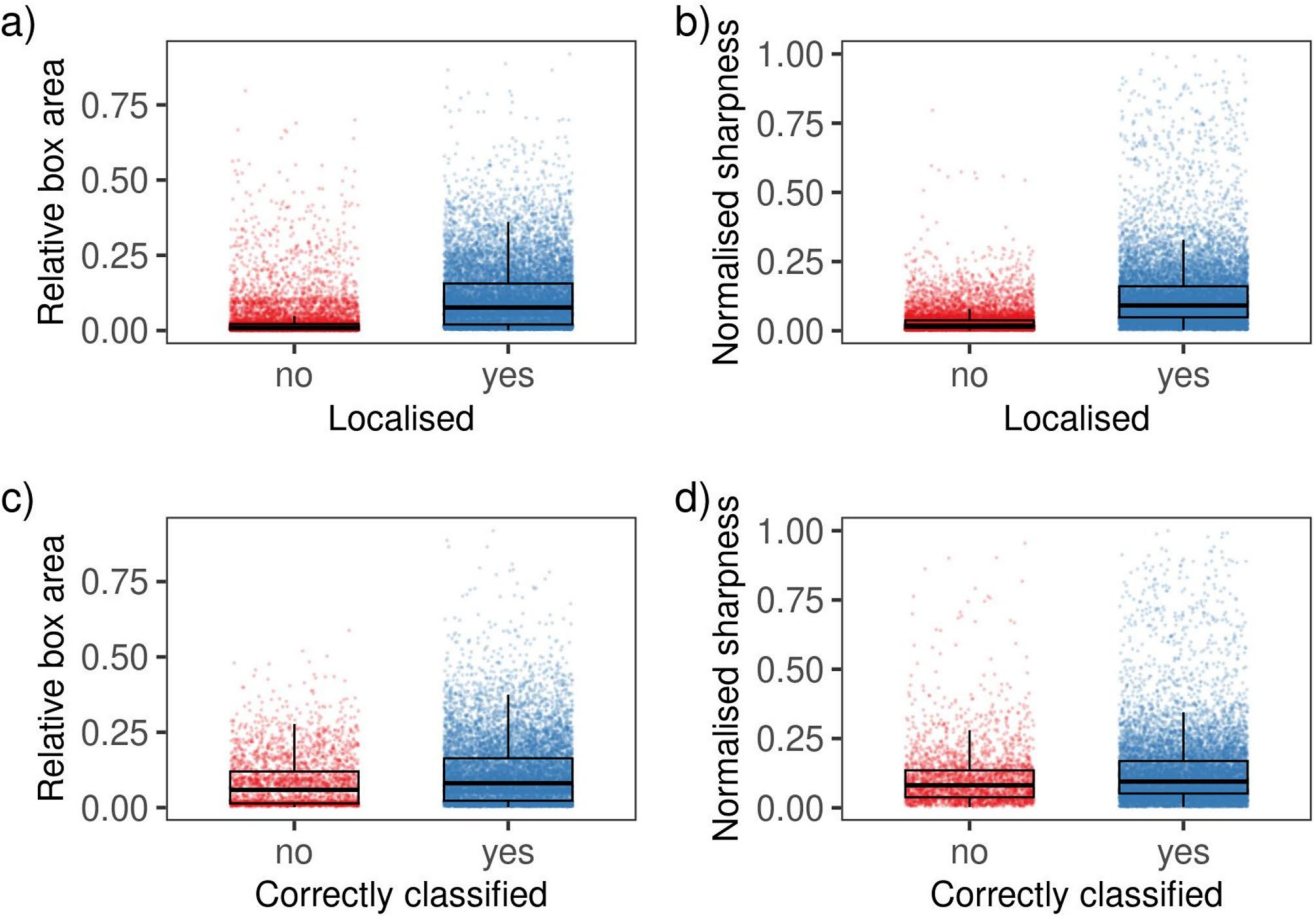
Boxplots displaying the distributions and interquartile ranges for relative bounding box area and normalised image sharpness (within the bounding box), categorised by successful localisation (eval-IoU = 0.5) and classification status (‘no’ vs. ‘yes’), across all arthropod categories in independent frames.

Smaller bounding boxes tended to contain blurrier arthropods (Spearman’s rank correlation ρ = 0.79, *p* < 0.05). Distributions of both box area and sharpness were long-tailed, with most arthropods appearing small and less sharp (Fig. 4a-d). Of 24,656 ground truth boxes, 14,654 (59.4%) were localised (eval-IoU = 0.5), while 10,002 (40.6%) remained undetected. Correctly localised arthropods had significantly larger bounding boxes (Δ_means_ = 0.0781, *p* < 0.05; Δ_medians_ = 0.0672, *p* < 0.05) and higher image sharpness (Δ_means_ = 0.0916, *p* < 0.05; Δ_medians_ = 0.0729, *p* < 0.05) compared to those not localised (Fig. 4a, b). Among localised arthropods, correctly classified instances also showed greater size (Δ_means_ = 0.0304, *p* < 0.05; Δ_medians_ = 0.0219, *p* < 0.05) and sharpness (Δ_means_ = 0.0230, *p* < 0.05; Δ_medians_ = 0.0133, *p* < 0.05) than misclassified ones (Fig. 4c, d).

**Fig. 5:**
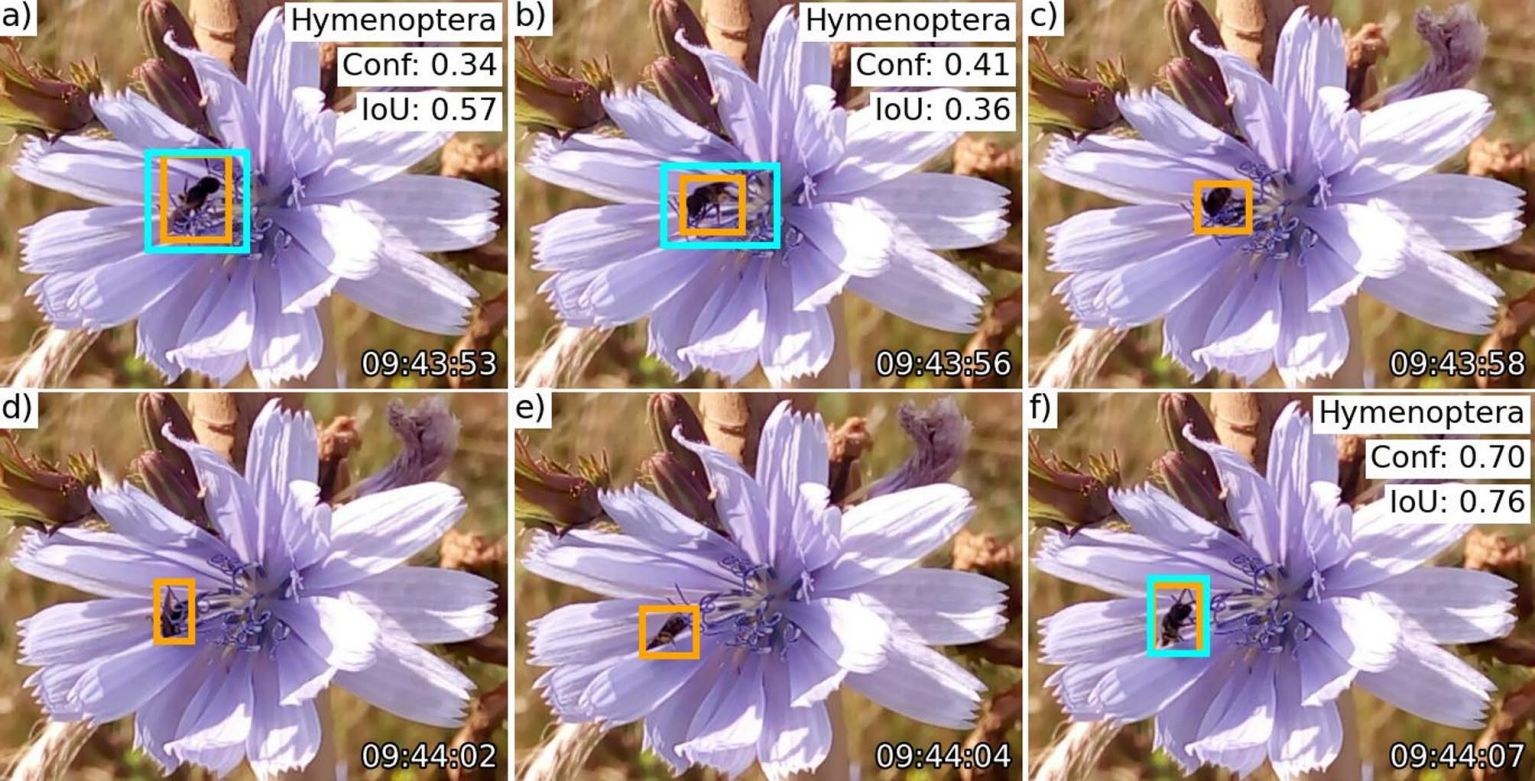
Example of arthropod presence across sequential time-lapse images, demonstrating overall sequence localisation even when partially obscured by flower parts (e.g., panels c–e). Localisation is considered successful when at least one ground truth box (orange) in the sequence achieves an IoU ≥ eval-IoU (0.5) with a predicted box (cyan), regardless of classification. Panels a, b, and f show correctly labelled Hymenoptera predictions, YOLO confidence scores (Conf.), and IoU values between ground truth and predictions. At eval-IoU 0.5, the predicted box in panel b is a false positive, but at eval-IoU 0.1, it is a true positive. The time stamp (bottom right corner of each panel) is provided in hh: mm: ss format.

For individual arthropod localisation within sequences, the optimised YOLOv5-small model achieved rates of 91.21% for Hymenoptera, 80.69% for Diptera, and 56.10% for OtherT flower visitors at eval-IoU 0.5. (Table 2). While this 0.5 threshold is commonly used, lowering it to 0.1 resulted in a marginal performance improvement (Supplementary Table S2). A localisation example in sequential time-lapse images is shown in Fig. 5. Classification recall was highest for Hymenoptera (*R* = 80.45%), followed by Diptera (*R* = 66.90%) and OtherT flower visitors (*R* = 47.97%), but accuracy ranked these groups in the opposite order (Table 2).

**Table 2:** Performance metrics of the optimised YOLOv5-small model for individual arthropod localisation and classification at eval-IoU 0.5. An arthropod represents a sequence of bounding boxes in time-lapse images. Columns report total individuals (N. ind.), mean relative bounding box area (Rel. B.box area), mean normalised sharpness (Norm. sharp.), localised arthropods (N), localisation recall or rate (R), classification precision (P), recall (R), F1 score and accuracy (Acc.). Confusion matrix results in “Predictions” show percentages (from N. ind.) and counts for Hymenoptera (Hym.), Diptera (Dip.), other arthropods (OtherT), and background/false negatives (Bg./FN).

Arthropod category	*N*. ind.	Rel. b.box area	Norm. sharp.	Localisation		Classification				Predictions - % and (counts)			
				*N*	*R*	*P*	*R*	F1	Acc.	Hym.	Dip.	OtherT	Bg./FN
Hymenoptera	1,013	0.1073	0.1020	924	0.9121	0.9772	0.8045	0.8825	0.8306	80.45% (815)	8.49% (86)	2.27% (23)	8.79% (89)
Diptera	145	0.0708	0.1178	117	0.8069	0.5243	0.6690	0.5879	0.8938	7.59% (11)	66.90% (97)	6.21% (9)	19.31% (28)
OtherT	123	0.0115	0.0321	69	0.5610	0.6484	0.4797	0.5514	0.9251	6.50% (8)	1.63% (2)	47.97% (59)	43.90% (54)
Overall	1,281	0.0751	0.0871	1,110	0.8665	0.8944	0.7580	0.8205	0.8468	-	-	-	-

The model correctly classified 815/1,013 (80.45%) Hymenoptera and 97/145 (66.90%) Diptera individual arthropods. Notably 86 (8.49%) Hymenoptera were identified as Diptera and 11 (7.59%) Diptera as Hymenoptera (Table 2). This bidirectional Hymenoptera-Diptera misclassification was evident in independent frames, with 76.96% of all misclassified Hymenoptera instances (boxes) labelled as Diptera and 64.88% of misclassified Diptera as Hymenoptera, significantly exceeding chance (*p* < 0.05, exact binomial test, expected probability 1/7 = 14.29%, Supplementary Table S3).

**Fig. 6:**
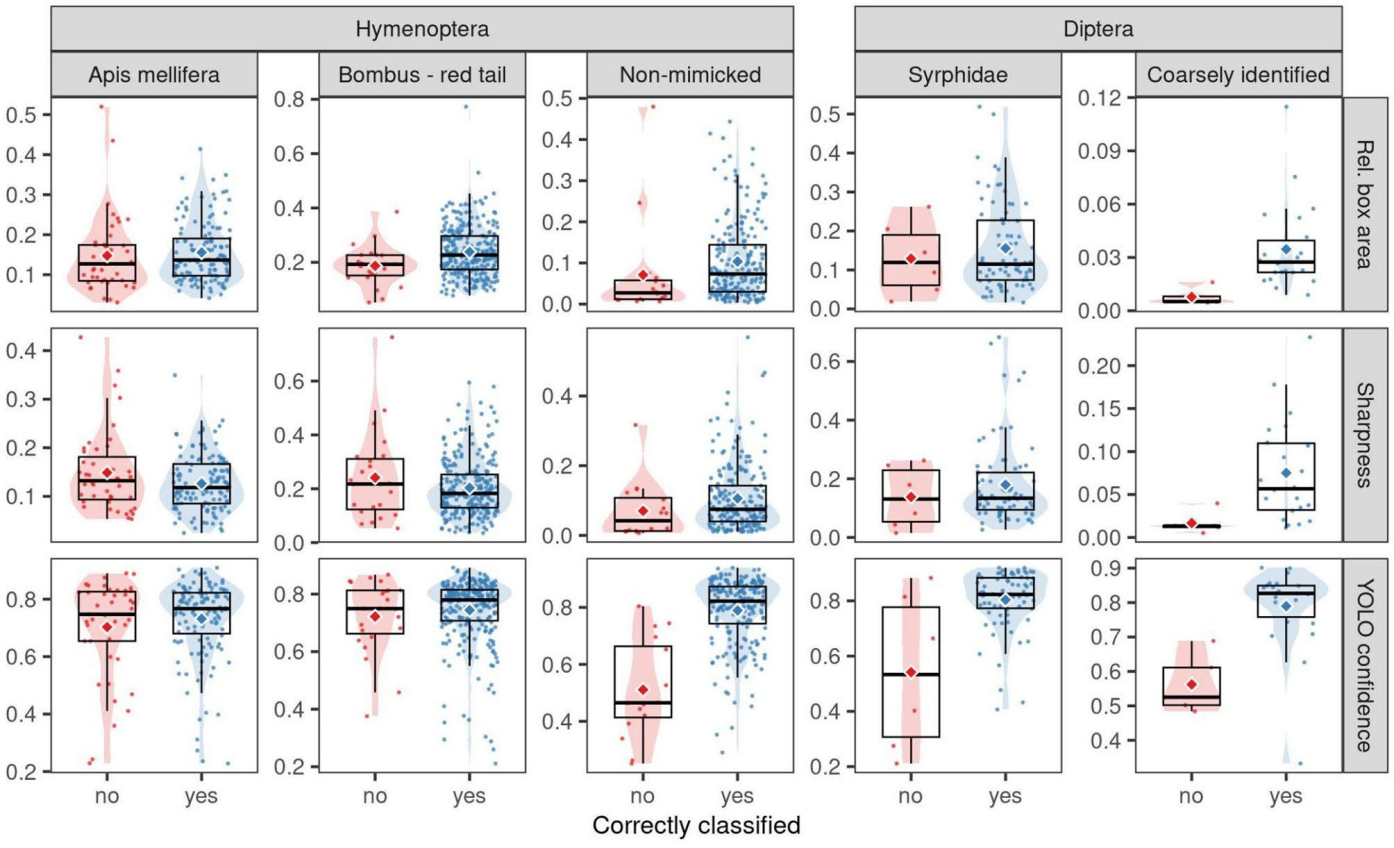
Boxplots showing distributions of relative bounding-box area, normalised image sharpness (within the bounding box), and model confidence score (YOLOv5-small) for pollinator taxa in Hymenoptera and Diptera orders, grouped by classification outcome (‘yes’ = correctly classified, ‘no’ = misclassified as the other order). Means are indicated by large diamond symbols. Syrphidae (Diptera) are known to mimic Hymenoptera such as *Apis mellifera* and red-tailed *Bombus*. For a detailed list of taxa in “Non-mimicked” and “Coarsely identified” (no family level ID) groups, see Supplementary Tables S4 and S5.

Of the 86 Hymenoptera individuals misclassified as Diptera, 43 were *Apis mellifera* (50.00%), 23 were red-tailed *Bombus* (26.74%), 8 were Halictidae (9.30%), and 8 were other Hymenopteran taxa (9.30%). The latter two groups (referred to as “non-mimicked”) are not targeted by Syrphidae mimicry. *Apis mellifera*, red-tailed *Bombus*, and Halictidae were common in the OOD dataset, collectively accounting for 56.47% (572/1,013) of Hymenoptera individuals, and thus we expected higher total misclassifications due to their higher total abundance in the dataset. However, from all misclassified Hymenoptera individuals (to Diptera or OtherT), the ones to Diptera exceeded chance (*p* < 0.05; exact binomial test; expected: 50% Diptera, 50% OtherT) for *Apis mellifera* (43/46, 93.48%) and red-tailed *Bombus* (23/26, 88.46%), but not for Halictidae (8/12, 66.67%) or Halictidae plus other non-mimicked taxa (“Non-mimicked”, 16/24, 66.67%; *p* > 0.05, see also Supplementary Table S4).

For *Apis mellifera*, cases misclassified as Diptera showed no significant differences in means or medians for bounding box area, sharpness, or model confidence from correctly classified ones (*p* > 0.05). Red-tailed *Bombus* misclassifications had significantly smaller mean bounding box areas (*p* < 0.05), but similar medians, sharpness, and model confidence (*p* > 0.05). In contrast, non-mimicked Hymenoptera misclassified as Diptera showed significantly higher confidence in correct classifications (*p* < 0.05). Misclassifications did not show significant differences in area or sharpness (*p* > 0.05; Fig. 6, Supplementary Table S4).

Among the 11 Diptera that were misclassified as Hymenoptera, six were Syrphidae and five were individuals that could not be identified by experts to the family level from the image (referred to hereafter as coarsely identified Diptera). The proportion of misclassifications as Hymenoptera did not differ from chance for either Syrphidae (66.67%) or coarsely identified (45.45%) Diptera (*p* > 0.05). Syrphidae misclassified as Hymenoptera showed no significant differences in bounding box area or sharpness from correctly classified cases (*p* > 0.05). Conversely, the misclassifications for coarsely identified Diptera were significantly smaller and blurrier than the correct classifications (*p* < 0.05). In both Diptera groups, model confidence was significantly higher for correct classifications (*p* < 0.05; Fig. 6, Supplementary Table S5).

## Discussion

Our results show that the optimised YOLOv5-small model, trained on citizen science images, correctly localised 91.21% and classified 80.45% of Hymenoptera individuals, as well as localized 80.69% and classified 66.90% of Diptera individuals. Detection performance was weaker for other flower visitors (OtherT), which were typically smaller and blurrier. However, their higher accuracy (92.51%) shows the model mislabels Hymenoptera or Diptera as OtherT less frequently.

To meet the demands of real-world pollinator monitoring, we chose lightweight models, as they promise energy-efficient deployment in field settings. Among those tested, YOLOv5-small, with the highest parameter count, outperformed others in F1 score, aligning with prior findings that greater model capacity (i.e., more trainable parameters) enhances performance^26,54^a trend also observed in our previous study^32^. Future work could explore higher-capacity architectures compatible with in situ hardware constraints. A critical consideration for on-device deployment is inference (prediction) time, particularly rapid inference being indispensable for real-time tracking to accurately estimate visitor numbers per target flower. For example, Sittinger et al.^55^ reported a maximum attainable inference time of 49 frames per second (approximately 0.02 sec. per image) for a single-class YOLOv5-nano detector (“blob” format) running at a 320 × 320 image resolution on an autonomous camera with a dedicated GPU, specifically for tracking insects landing on a platform. Since image resolution impacts inference time, our models, though trained for a 640 × 640 resolution, could be retrained and converted to run inference at 320 × 320, potentially achieving similar performance on such custom camera hardware. For devices without a dedicated GPU, such as those equipped solely with CPUs, inference times are longer. Our previous work^32^ reported estimates for inference times (localisation and classification in one step) on a single core of a AMD EPYC 7551P 2.0 GHz CPU (within a server) for a 640 × 640 input resolution: YOLOv5-nano processed an image in 0.1893 sec., while YOLOv5-small took 0.4833 sec. per image (“PyTorch” format). Although a field device’s CPU would be less powerful and it would also handle essential tasks like image capture and operating system functions, reducing effective inference speed, future studies could test if these models can be adapted (e.g., via pruning and quantization^56^ to run in the background or overnight on CPU-based systems to filter out images devoid of arthropods, exploring viable solutions for large-scale data pre-processing.

The grid search NMS optimisation, maximising the F1 score of arthropod detectors on the unseen OOD image dataset under complex field conditions, has practical implications for camera system design. For instance, adapting Sittinger et al.’s^55^ setup for monitoring flower visitors could enhance on-device detection performance beyond default NMS values. This optimisation reflects dataset-specific tuning, as evidenced by comparing prior and current studies. In our earlier work with citizen science test images^32^a higher NMS-IoU suited dense, overlapping bounding boxes of ants and bugs (e.g., images near ant colonies). Conversely, the OOD flower-visit dataset, dominated by images containing single arthropods, favoured a lower NMS-IoU, with performance declining at higher values (Fig. 3a, c). A higher NMS-IoU threshold permits overlapping boxes, aiding detection of closely spaced arthropods, whereas a lower threshold enhances precision by minimising redundant predictions for solitary arthropods.

Our pollinator localisation tests have practical implications, demonstrating the potential of object detection models trained on citizen science images to assist in annotating time-lapse field datasets, where most frames lack arthropods (e.g., over 90%^37^^57^^58^). Even by enabling a single prediction per sequence, these models could allow annotators to target relevant frames, bypassing manual review of arthropod-free images. Manual annotation of a 460,056-image time-lapse dataset previously required approximately 1,000 hours^37^whereas the YOLOv5-small model, performing both localization and classification, processed 23,899 OOD images in 419 sec. (~ 0.0175 sec. per image) on an NVIDIA RTX A6000 GPU, a desktop-grade component, suggesting around 2.24-hours runtime for the larger dataset, assuming fast image access. However, we noted that false positive (FP) rates on OOD images, including floral-only backgrounds, surpassed those on citizen science images, which more closely resemble the training set^32^. Our primary evaluation utilised an eval-IoU threshold of 0.5, consistent with standard practice^59^ and our previous work^32^as this threshold emphasizes the precise localisation of arthropods. Nevertheless, we observed that allowing larger predicted bounding boxes with using sub-0.5 IoU (e.g., Fig. 5) could enhance overall localisation and reduce FPs (e.g., results at eval-IoU 0.1 in Supplementary Table S2). This suggests that a lower eval-IoU may be beneficial when prioritizing the localisation of arthropods over highly accurate bounding box alignment. To further reduce FP rates and improve precision, including floral backgrounds without pollinators in training may prove beneficial. Another challenge is that smaller, less sharp arthropods are more likely to be missed. While the model effectively localised larger, common Hymenoptera and Diptera pollinators, it struggled with other flower visitors in the OOD dataset, which tended to be smaller and blurrier.

After localisation, classifying flower visitors challenged the model more, with significant bidirectional Hymenoptera and Diptera misclassifications outnumbering those to other categories, alongside reduced performance for other arthropods. While it distinguished these categories effectively on in-distribution images^32^this proficiency declined on the OOD dataset, where arthropods were on average 4.5 times smaller than in-distribution counterparts and sometimes occluded by flower parts (e.g., Fig. 5). This aligns with studies reporting reduced generalisation on organisms across new locations, time-frames, and sensors^27,31,57,60–62^alongside pollinator-specific occlusion challenges^63–65^. Moreover, the pretrained models were not trained with more images of either Hymenoptera or Diptera than other categories, ruling out dataset bias as a cause of cross-order misclassifications. This is further supported by the fact that, despite Lepidoptera being the majority class (nearly twice as abundant) in the training data^32^the model was robust against this class imbalance and rarely mislabelled Hymenoptera (the majority class in the OOD test set) or Diptera (the second most abundant class) as Lepidoptera (e.g., Supplementary Table S3). Likewise, the higher accuracy for OtherT flower visitors shows the model less often mislabels Hymenoptera or Diptera as OtherT.

Given these, Syrphidae mimicry most likely exacerbates the significant Hymenoptera-Diptera confusion, with syrphids like *Eristalis* spp. and *Volucella bombylans* resembling bees (e.g., *Apis mellifera*^66^ and red-tailed *Bombus* (e.g., *B. lapidarius*, *B. pratorum*^67^, respectively, mimicking their warning signals to deter predators. In the OOD dataset, larger or sharper arthropod instances exhibited significantly distinct distributions from smaller or blurrier counterparts for both localisation and classification. However, *Apis mellifera* and red-tailed *Bombus*, misclassified as Diptera, were as large and sharp as correctly classified cases, and the model was equally confident in these misclassifications most likely due to mimicry. In contrast, cross-order misclassified taxa not mimicked by Syrphidae (e.g., Halictidae, Cynipidae in Hymenoptera) and a few small, coarsely identified Diptera, had significantly higher model confidence in correct classifications. Their misclassified cases tended to be smaller and blurrier than correctly classified ones, likely explaining the mislabelling. Syrphidae misclassified as Hymenoptera were as large and sharp as correctly classified cases, but the model was significantly less confident in misclassifications. While these results might suggest that mimicry confuses the model more in one direction, with mimicked Hymenoptera more likely to be misclassified as Diptera than mimicking Syrphidae as Hymenoptera, we cannot say this conclusively due to the smaller sample size of Syrphidae individuals that were misclassified as Hymenoptera.

To improve localisation and classification, we consider several steps for future research. First, integrating citizen-science and field images, as in recent studies^68,69^would enhance model generalisation for real-world pollinator monitoring using time-lapse photography. Given that multiple studies have highlighted the scarcity of annotated field datasets for small arthropods, including pollinators^23,25,70,71^our study addresses this gap by providing the OOD dataset (cropped and full-frame images) for training arthropod detectors for custom field cameras. Our OOD dataset provides complex floral backgrounds, reflecting the variability inherent in automated pollinator monitoring, where images are captured passively with a fixed smartphone setup, without real-time human selection, curation or framing. The OOD dataset is however characterised by a natural class imbalance, with the majority class represented by Hymenoptera, followed by Diptera. Therefore, models trained with this dataset should be deployed at locations where similar arthropod distributions are expected. Fortunately, Hymenoptera and Diptera are common orders of pollinators in Europe, often dominating sampled plant-pollinator networks^72^. Class imbalance is nevertheless a source of bias and this could be mitigated by sampling underrepresented classes from available citizen science sources and/or applying more data augmentation on those classes. At the same time, maintaining a clear separation between training and test sets is essential because time-lapse image sequences can introduce a risk of data leakage^73,74^ if highly similar frames are split between these sets, potentially inflating model performance. In such cases, the network may rely on shortcut learning^28^recognising near-identical images based on superficial visual similarities (e.g., background patterns, nearly identical insect poses) rather than developing a truly generalisable understanding of arthropod features. To mitigate this, careful dataset partitioning is needed to prevent the model from exploiting temporal redundancies (e.g., highly similar consecutive frames depicting the same individual arthropod should be kept within a single set, either training, validation, or test, rather than split across them).

Second, model performance could improve through a two-steps approach, as suggested in other studies^55,57,62,68,75^. For example, an initial single-class object detector, such as YOLO^76^could localise arthropods (e.g., arthropod vs. background), followed by a classifier to identify their cropped images at finer taxonomic levels. In this study, the predicted labels were disregarded for the purpose of the arthropod localisation task, in line with our objective to develop a generic single-class arthropod detector. This two-steps approach also allows the community to choose object detectors suited to their field hardware while leveraging diverse classification methods in post-processing, such as region-specific classifiers trained on continuously expanding datasets^77^taxon-specific classifiers^78^ (that can be applied at specific locations or time frames to accommodate class imbalance due to natural variation), large multimodal models^79^or hierarchical classification via custom classifier^68,80,81^ and vision foundation models capable of learning hierarchical representations^82^. Furthermore, integrating object detection with segmentation has been shown to improve bumblebee species identification by removing noisy backgrounds and focusing classifiers on the most relevant features^83^. Additionally, citizen science platforms encourage users to upload cropped images of organisms^34^providing a rich source of training data for such classifiers. Another advantage is the potential for multi-view classification^84^leveraging sequential images of the same arthropod. Similar to how taxonomists examine multiple frames (e.g., Fig. 5) to improve identification despite occlusions or lower-quality frames, a multi-view CNN could refine predictions. In our study, we simplified this by assigning the label with the highest confidence score across a sequence, but a dedicated multi-view CNN could further enhance performance.

Third, preprocessing time-lapse images to highlight arthropod features against the background^63^ could enhance localisation if compatible with low-energy field cameras, or, if too energy-intensive, applied later on stored images rather than in real-time.

Fourth, our results confirm arthropod size and image sharpness as important factors to localisation and classification, aligning with Nguyen et al.’s^70,85^ findings on small-object detection challenges. The correlation between size and sharpness indicates also that arthropods further from the camera, or small arthropods in general, are most likely to be out of focus. Optimising image capture thus involves defining a region of interest and focusing on the target flower or inflorescence segment within, to maximise arthropod size in the frame. The region of interest can be defined via flower detection, segmentation, or pre-defined at the start of the recording session. This also aligns with future research where we aim to develop custom cameras based on the technology proposed by Sittinger et al.^55^that focus solely on target flowers, discarding noisy backgrounds that may contain out-of-focus flowers or cluttered patches of vegetation, which could confuse the models. Fixed focus is also crucial, and we adopted it when collecting the OOD dataset to prevent autofocus from shifting to background and blurring arthropods, as observed by Bjerge et al.^63^. Additionally, including blurred images in training datasets could further improve generalisation, as shown in larval fish detection^86^.

Lastly, tiling full-frame images for detection could improve small-object localisation^87,88^ by preserving details without downscaling to detector’s resolution. However, sliced inference like SAHI^87^ increases computational demands on low-power field devices. While not our primary focus, our preliminary SAHI test with YOLOv5-small on the OOD dataset showed slight F1 gains, but increased false positives and processing time (Supplementary Table S6). Still, fine-tuning SAHI could aid annotation of high-resolution time-lapse datasets when real-time processing is not required.

Implementing these proposed steps could enhance the detection of flower visitors, thereby facilitating the tracking of individual pollinators and enabling estimates of floral visit abundance, a key goal for automated pollinator monitoring. Examples of insect tracking can be found in recent studies^55,64,89^.

## Conclusion

Our findings highlight the potential and limitations of lightweight YOLO detector models, trained on citizen science images, for localising and classifying flower visitors in out-of-distribution (OOD) time-lapse field images captured with a fixed smartphone setup. Localisation was generally effective for common Hymenoptera and Diptera pollinators, defined as cases where at least one bounding box in a time-lapse sequence was correctly placed. However, classification proved more challenging, impacted by arthropod size, image sharpness, and mimicry between Syrphidae (Diptera) and Hymenoptera. Smaller, blurrier arthropods, including less common flower visitors, were harder to detect, and the increase in false positives compared to in-distribution data revealed limitations when generalising to complex field conditions.

These results have practical value for pollinator monitoring, showing potential for automating annotation of common Hymenoptera and Diptera pollinators in large time-lapse datasets, likely easing manual workloads. Future work could enhance performance by combining field and citizen science images in training, using a two-step detection-classification approach, optimising image capture to enhance arthropod size and sharpness, or adjusting NMS-IoU for specific ecological contexts. By providing an OOD dataset and identifying key challenges, this work contributes to the development of more robust machine learning tools for pollinator monitoring in natural environments.

## Supplementary Information

Below is the link to the electronic supplementary material.

Supplementary Material 1

## Data Availability

The image dataset related to this research is available at https://doi.org/10.5281/zenodo.15096609. The open-source code for the experiments is hosted on GitHub at https://github.com/valentinitnelav/smartphone-insect-detect.
