# Supplementary material for: Successes and limitations of pretrained YOLO detectors applied to unseen time-lapse images for automated pollinator monitoring
Source: Sci Rep. 2025 Aug 21;15:30671. doi: 10.1038/s41598-025-16140-z (PMC12368093; doi:10.1038/s41598-025-16140-z)
Supplement: Supplementary file 1 — Supplementary Material 1 [file 41598_2025_16140_MOESM1_ESM.docx]

**Supplementary Information**

Successes and limitations of pretrained YOLO detectors applied to unseen time-lapse images for automated pollinator monitoring

Valentin Ștefan^1,2,3,*^, Thomas Stark^4^, Michael Wurm^4^, Hannes Taubenböck^4,5^, Tiffany M. Knight^1,2,3,6^

^1^Department of Species Interaction Ecology, Helmholtz Centre for Environmental Research - UFZ, Leipzig, Germany.

^2^German Centre for Integrative Biodiversity Research (iDiv) Halle-Jena-Leipzig, Leipzig, Germany.

^3^Institute of Biology, Martin Luther University Halle-Wittenberg, Halle (Saale), Germany. ^4^German Remote Sensing Data Center (DFD), German Aerospace Center (DLR), Oberpfaffenhofen, Germany.

^5^Department of Global Urbanisation and Remote Sensing, University of Würzburg, Würzburg, Germany.

^6^Department of Science and Conservation, National Tropical Botanical Garden, Kalāheo, USA

*Corresponding author: valentin.stefan@idiv.de

# Abstract

Pollinating insects provide essential ecosystem services, and using time-lapse photography to automate their observation could improve monitoring efficiency. Computer vision models, trained on clear citizen science photos, can detect insects in similar images with high accuracy, but their performance in images taken using time-lapse photography is unknown. We evaluated the generalisation of three lightweight YOLO detectors (YOLOv5-nano, YOLOv5-small, YOLOv7-tiny), previously trained on citizen science images, for detecting ~1,300 flower-visiting arthropod individuals in nearly 24,000 time-lapse images captured with a fixed smartphone setup. These field images featured unseen backgrounds and smaller arthropods than the training data. YOLOv5-small, the model with the highest number of trainable parameters, performed best, localising 91.21% of Hymenoptera and 80.69% of Diptera individuals. However, classification recall was lower (80.45% and 66.90%, respectively), partly due to Syrphidae mimicking Hymenoptera and the challenge of detecting smaller, blurrier flower visitors. This study reveals both the potential and limitations of such models for real-world automated monitoring, suggesting they work well for larger and sharply visible pollinators but need improvement for smaller, less sharp cases.

# Supplementary Materials

## **Supplementary Methods**

The standard detection approach used by the tested YOLO models relies on a technique called non-maximum suppression (NMS). This method operates independently of the ground-truth bounding boxes and helps the detector to eliminate multiple, similar predictions for the same target object, aiming to produce a single, accurate prediction. Specifically, it allows suppressing predicted boxes that substantially overlap with the predicted box having the highest prediction confidence score (e.g., Supplementary Fig. S1). NMS, and therefore the detector’s performance, is sensitive to two parameters that impose a trade-off between precision and recall: NMS confidence score and NMS Intersection over Union (NMS-IoU).

To optimise localisation performance, we fine-tuned the NMS parameters to maximise the F1 score (harmonic mean of precision and recall). Specifically, we ran detections on the OOD dataset using a near-zero (0.001) NMS confidence score and varied the NMS-IoU threshold from 0.1 to 0.9 in 0.1 steps. The low NMS-confidence threshold allowed a broad range of predictions to be considered, each with an associated prediction confidence score. Post-detection, we classified predicted boxes as true positives (TP), false positives (FP), or false negatives (FN) based on an evaluation Intersection over Union (eval-IoU) threshold (e.g., 0.5 and 0.1). Further, we computed precision, recall, F1 score across prediction confidence scores (F1-confidence curve), and the area under the precision-recall curve (AUC, also known as average precision). We repeated this evaluation for each NMS-IoU threshold, using the maximum attainable F1 score (from the F1-confidence curves) to identify the best model and its optimal NMS parameters.

To evaluate model efficacy for the localisation of boxes with arthropods in independent frames (within a given image, regardless of the time-lapse sequence), specific criteria were adopted (see also Supplementary Fig. S2):

- True Positive (box-TP): defined as a predicted bounding box that is adequately paired with a ground truth box, considering only those pairs for which the eval-IoU exceeds or equals a threshold (e.g., 0.5). When multiple such predicted boxes are present, the box with the highest YOLO confidence score is selected, irrespective of its predicted label.
- False Positive (box-FP): defined as a predicted bounding box that does not correspond to any ground truth box (model “hallucinates”), or when it does correspond but has an eval-IoU score below the threshold (insufficient overlap). Essentially this is an instance where the model inaccurately localises an arthropod (type I error).
- False Negative (box-FN): defined as a ground truth box without any corresponding predicted box (type II error). This situation occurs when the model fails to place any predicted bounding boxes in an image containing an arthropod or they are all box-FPs as defined above.

| **Model** | **NMS-IoU** | **NMS confidence** | **Max. F1** | **Max. AUC** | **Nr. parameters (million)** |
| --- | --- | --- | --- | --- | --- |
| YOLOv5-small | 0.3 | 0.2019 | 0.7019 | 0.6497 | 7.2 |
| YOLOv7-tiny | 0.3 | 0.2236 | 0.6617 | 0.6294 | 6.2 |
| YOLOv5-nano | 0.1 | 0.1648 | 0.6539 | 0.6111 | 1.9 |

## **Table S1.** Optimal values for the NMS hyperparameters IoU and confidence that maximised F1 score (Max. F1) and area under the precision-recall curve (Max. AUC) for each YOLO model. Results correspond to the bounding box localisation task (localisation of arthropod instances in independent frames). Optimisation was run with a NMS confidence score of 0.001 to obtain the F1 curves.

| **Arthropod category** | **N. ind.** | **Localisation** | | **Classification** | | | | **Predictions - % and (counts)** | | | |
| --- | --- | --- | --- | --- | --- | --- | --- | --- | --- | --- | --- |
|  |  | **N** | **R** | **P** | **R** | **F1** | **Acc.** | **Hym.** | **Dip.** | **OtherT** | **Bg./FN** |
| Hymenoptera | 1,013 | 941 | 0.9289 | 0.9762 | 0.8105 | 0.8857 | 0.8345 | 81.05%  (821) | 8.79%  (89) | 3.06%  (31) | 7.11%  (72) |
| Diptera | 145 | 123 | 0.8483 | 0.5260 | 0.6966 | 0.5994 | 0.8946 | 6.90%  (10) | 69.66%  (101) | 8.28%  (12) | 15.17%  (22) |
| OtherT | 123 | 72 | 0.5854 | 0.5825 | 0.4878 | 0.5310 | 0.9173 | 8.13%  (10) | 1.63%  (2) | 48.78%  (60) | 41.46%  (51) |
| Overall | 1,281 | 1,136 | 0.8868 | 0.8874 | 0.7666 | 0.8226 | 0.8493 | - | - | - | - |

## **Table S2.** Performance metrics of the best-selected model for individual arthropod localisation and classification at eval-IoU of 0.1. See Table 2 in the manuscript for abbreviations.

| **Arthropod category** | | **N. miscl. Instances** | **Total miscl. instances** | **% miscl.** | **N. miscl. expected** | **p-value** |
| --- | --- | --- | --- | --- | --- | --- |
| **Ground truth** | **Misclassified to:** |  |  |  |  |  |
| Diptera | Araneae | 2 | 467 | 0.43 | 67 | 1.0000 |
| Diptera | Coleoptera | 67 |  | 14.35 | 67 | 0.5050 |
| Diptera | Hemiptera | 19 |  | 4.07 | 67 | 1.0000 |
| **Diptera** | **Hymenoptera** | **303** |  | **64.88** | **67** | **< 0.0001** |
| Diptera | Hymenoptera - Formicidae | 11 |  | 2.36 | 67 | 1.0000 |
| Diptera | Lepidoptera | 65 |  | 13.92 | 67 | 0.6096 |
| Hymenoptera | Araneae | 10 | 1,710 | 0.58 | 244 | 1.0000 |
| Hymenoptera | Coleoptera | 172 |  | 10.06 | 244 | 1.0000 |
| **Hymenoptera** | **Diptera** | **1,316** |  | **76.96** | **244** | **< 0.0001** |
| Hymenoptera | Hemiptera | 16 |  | 0.94 | 244 | 1.0000 |
| Hymenoptera | Hymenoptera - Formicidae | 120 |  | 7.02 | 244 | 1.0000 |
| Hymenoptera | Lepidoptera | 74 |  | 4.33 | 244 | 1.0000 |
| Hymenoptera | Orthoptera | 2 |  | 0.12 | 244 | 1.0000 |

## **Table S3.** Misclassification frequencies of Diptera and Hymenoptera ground truth instances (bounding boxes) in independent frames, showing the observed count and percentage of misclassifications (miscl.) compared to the expected values under random chance. Expected probability = 1/7, with seven possible misclassifications per category. The one-tailed exact binomial test assessed whether these misclassifications occurred significantly more often than expected by chance, specifically testing for a higher frequency. Significant misclassifications (p < 0.05) are highlighted in bold.

| **Type** | **#** | **Variables** | ***Apis mellifera*** | ***Bombus* red tail** | **Not mimicked** |
| --- | --- | --- | --- | --- | --- |
| N. individuals and binomial test of misclassification rates | 1 | N. individuals in dataset | 185 | 301 | 259 |
|  | 2 | N. localised | 177 | 291 | 229 |
|  | 3 | N. correctly classified | 131 | 265 | 205 |
|  | 4 | N. misclassified, total (from those localised) | 46 | 26 | 24 |
|  | 5 | N. misclassified as Diptera | 43 | 23 | 16 |
|  | 6 | N. misclassified as OtherT | 3 | 3 | 8 |
|  | 7 | % misclassified as Diptera from #4 | 93.48 | 88.46 | 66.67 |
|  | 8 | N. misclassified as Diptera, expected | 23 | 13 | 12 |
|  | 9 | p-value, binomial test for #5 & #7 | < 0.001 | < 0.001 | 0.0758 |
|  | 10 | p-value, is significant, for #9 (< 0.05?) | yes | yes | no |
| Relative bounding box area | 11 | Mean relative b.box area for #5 | 0.1477 | 0.1871 | 0.0711 |
|  | 12 | S.D. for #11 | 0.0966 | 0.0734 | 0.1234 |
|  | 13 | Mean relative b.box area for #3 | 0.1556 | 0.2385 | 0.1036 |
|  | 14 | S.D. for #13 | 0.0748 | 0.0923 | 0.0949 |
|  | 15 | Difference means #13 - #11 | 0.0079 | 0.0514 | 0.0325 |
|  | 16 | Permutation quantile conf. interval for #15 | -0.0301:0.0279 | -0.0421:0.0349 | -0.0538:0.0458 |
|  | 17 | p-value, permutations for #15 | 0.611 | 0.005 | 0.219 |
|  | 18 | p-value is significant, for #15 (< 0.05?) | no | yes | no |
|  | 19 | Median relative b.box area for #5 | 0.127 | 0.1927 | 0.0274 |
|  | 20 | Median relative b.box area for #3 | 0.1366 | 0.2269 | 0.0733 |
|  | 21 | Difference medians #20 - #19 | 0.0096 | 0.0343 | 0.0459 |
|  | 22 | Permutation quantile conf. interval for #21 | -0.0363:0.0322 | -0.0486:0.0425 | -0.0699:0.0397 |
|  | 23 | p-value, permutations for #21 | 0.565 | 0.122 | 0.083 |
|  | 24 | p-value is significant, for #21 (< 0.05?) | no | no | no |
| Normalised sharpness (Sobel - Tenengrad operator) | 25 | Mean normalised sharpness for #5 | 0.1487 | 0.2412 | 0.0702 |
|  | 26 | S.D. for #25 | 0.0833 | 0.165 | 0.0812 |
|  | 27 | Mean normalised sharpness for #3 | 0.1263 | 0.2036 | 0.1061 |
|  | 28 | S.D. for #27 | 0.0564 | 0.1055 | 0.0951 |
|  | 29 | Difference means #27 - #25 | -0.0224 | -0.0377 | 0.0359 |
|  | 30 | Permutation quantile conf. interval for #29 | -0.0239:0.0222 | -0.0483:0.0440 | -0.0521:0.0429 |
|  | 31 | p-value, permutations for #29 | 0.057 | 0.110 | 0.145 |
|  | 32 | p-value significant, for #29 (< 0.05?) | no | no | no |
|  | 33 | Median normalised sharpness for #5 | 0.1321 | 0.2175 | 0.042 |
|  | 34 | Median normalised sharpness for #3 | 0.118 | 0.1827 | 0.0749 |
|  | 35 | Difference medians #34 - #33 | -0.0141 | -0.0348 | 0.0329 |
|  | 36 | Permutation quantile conf. interval for #35 | -0.0272:0.0250 | -0.0491:0.0450 | -0.0531:0.0322 |
|  | 37 | p-value, permutations for #35 | 0.390 | 0.125 | 0.124 |
|  | 38 | p-value is significant, for #35 (< 0.05?) | no | no | no |
| YOLO confidence | 39 | Mean YOLO confidence for #5 | 0.7028 | 0.7219 | 0.5111 |
|  | 40 | S.D. for #39 | 0.1706 | 0.1279 | 0.1706 |
|  | 41 | Mean YOLO confidence for #3 | 0.7316 | 0.7443 | 0.7894 |
|  | 42 | S.D. for #41 | 0.1361 | 0.1149 | 0.1186 |
|  | 43 | Difference means #41 - #39 | 0.0287 | 0.0224 | 0.2783 |
|  | 44 | Permutation quantile conf. interval for #43 | -0.0455:0.0495 | -0.0476:0.0518 | -0.0675:0.0805 |
|  | 45 | p-value, permutations for #43 | 0.235 | 0.384 | < 0.001 |
|  | 46 | p-value is significant, for #43 (< 0.05?) | no | no | yes |
|  | 47 | Median YOLO confidence for #5 | 0.7472 | 0.7496 | 0.465 |
|  | 48 | Median YOLO confidence for #3 | 0.7669 | 0.7798 | 0.8222 |
|  | 49 | Difference medians #48 - #47 | 0.0197 | 0.0301 | 0.3571 |
|  | 50 | Permutation quantile conf. interval for #49 | -0.0461:0.0501 | -0.0309:0.0620 | -0.0525:0.0934 |
|  | 51 | p-value, permutations for #49 | 0.487 | 0.128 | < 0.001 |
|  | 52 | p-value is significant, for #49 (< 0.05?) | no | no | yes |

## **Table S4.** Quantitative comparison of correctly classified Hymenoptera and those misclassified as Diptera. The “Not mimicked” category includes 8 Halictidae cases, 2 *Halictus* (Halictidae), 3 Cynipidae, 1 Andrenidae, 1 *Megachile* (Megachilidae), and 1 identified only to Cynipoidea superfamily. Metrics were computed using values from the predicted boxes with the highest confidence, as these determined the final classification of an individual pollinator across frames.

| **Type** | **#** | **Variables** | **Syrphidae** | **Coarsely identified** |
| --- | --- | --- | --- | --- |
| N. individuals and binomial test of misclassification rates | 1 | N. individuals in dataset | 92 | 53 |
|  | 2 | N. localised | 84 | 33 |
|  | 3 | N. correctly classified | 75 | 22 |
|  | 4 | N. misclassified, total (from those localised) | 9 | 11 |
|  | 5 | N. misclassified as Hymenoptera | 6 | 5 |
|  | 6 | N. misclassified as OtherT | 3 | 6 |
|  | 7 | % misclassified as Hymenoptera from #4 | 66.67 | 45.45 |
|  | 8 | N. misclassified as Hymenoptera, expected | 4 | 6 |
|  | 9 | p-value, binomial test for #5 & #7 | 0.2539 | 0.7256 |
|  | 10 | p-value, is significant, for #9 (< 0.05?) | no | no |
| Relative bounding box area | 11 | Mean relative b.box area for #5 | 0.129 | 0.0078 |
|  | 12 | S.D. for #11 | 0.0932 | 0.0048 |
|  | 13 | Mean relative b.box area for #3 | 0.1557 | 0.0346 |
|  | 14 | S.D. for #13 | 0.1115 | 0.0243 |
|  | 15 | Difference means #13 - #11 | 0.0266 | 0.0268 |
|  | 16 | Permutation quantile conf. interval for #15 | -0.1103:0.0798 | -0.0285:0.0181 |
|  | 17 | p-value, permutations for #15 | 0.575 | 0.030 |
|  | 18 | p-value is significant, for #15 (< 0.05?) | no | yes |
|  | 19 | Median relative b.box area for #5 | 0.1192 | 0.0052 |
|  | 20 | Median relative b.box area for #3 | 0.1149 | 0.0274 |
|  | 21 | Difference medians #20 - #19 | -0.0043 | 0.0222 |
|  | 22 | Permutation quantile conf. interval for #21 | -0.1385:0.0574 | -0.0307:0.0145 |
|  | 23 | p-value, permutations for #21 | 0.924 | 0.036 |
|  | 24 | p-value is significant, for #21 (< 0.05?) | no | yes |
| Normalised sharpness (Sobel-Tenengrad operator) | 25 | Mean normalised sharpness for #5 | 0.1381 | 0.0168 |
|  | 26 | S.D. for #25 | 0.1056 | 0.0133 |
|  | 27 | Mean normalised sharpness for #3 | 0.1797 | 0.0751 |
|  | 28 | S.D. for #27 | 0.1396 | 0.0592 |
|  | 29 | Difference means #27 - #25 | 0.0416 | 0.0584 |
|  | 30 | Permutation quantile conf. interval for #29 | -0.1299:0.0902 | -0.0609:0.0497 |
|  | 31 | p-value, permutations for #29 | 0.522 | 0.037 |
|  | 32 | p-value significant, for #29 (< 0.05?) | no | yes |
|  | 33 | Median normalised sharpness for #5 | 0.1308 | 0.0134 |
|  | 34 | Median normalised sharpness for #3 | 0.1337 | 0.0567 |
|  | 35 | Difference medians #34 - #33 | 0.0029 | 0.0433 |
|  | 36 | Permutation quantile conf. interval for #35 | -0.1149:0.0551 | -0.0866:0.0370 |
|  | 37 | p-value, permutations for #35 | 0.916 | 0.127 |
|  | 38 | p-value is significant, for #35 (< 0.05?) | no | no |
| YOLO confidence | 39 | Mean YOLO confidence for #5 | 0.5417 | 0.5622 |
|  | 40 | S.D. for #39 | 0.2848 | 0.0858 |
|  | 41 | Mean YOLO confidence for #3 | 0.8034 | 0.7896 |
|  | 42 | S.D. for #41 | 0.1077 | 0.1228 |
|  | 43 | Difference means #41 - #39 | 0.2618 | 0.2274 |
|  | 44 | Permutation quantile conf. interval for #43 | -0.0889:0.1345 | -0.1244:0.1466 |
|  | 45 | p-value, permutations for #43 (< 0.05?) | < 0.001 | 0.002 |
|  | 46 | p-value is significant, for #43 | yes | yes |
|  | 47 | Median YOLO confidence for #5 | 0.5331 | 0.5252 |
|  | 48 | Median YOLO confidence for #3 | 0.8231 | 0.8266 |
|  | 49 | Difference medians #48 - #47 | 0.2899 | 0.3014 |
|  | 50 | Permutation quantile conf. interval for #49 | -0.0722:0.1054 | -0.0724:0.2088 |
|  | 51 | p-value, permutations for #49 | 0.001 | 0.003 |
|  | 52 | p-value is significant, for #49 (< 0.05?) | yes | yes |

## **Table S5.** Quantitative comparison of correctly classified Diptera and those misclassified as Hymenoptera. The “Coarsely identified” category includes 4 cases identified only to order level, and 1 identified only to Muscomorpha infraorder. Metrics were computed using values from the predicted boxes with the highest confidence, as these determined the final classification of an individual pollinator across frames.

| **Detection mode** | **TP** | **FP** | **FN** | **P** | **R** | **F1** | **AUC** | **Conf.** | **IoU** | **Overlap** | **Detection time, sec.** |
| --- | --- | --- | --- | --- | --- | --- | --- | --- | --- | --- | --- |
| YOLOv5s + SAHI | 15,282 | 2,912 | 9,556 | 0.8399 | 0.6153 | 0.7103 | 0.5849 | 0.5 | 0.5 | 0.2 | 951 |
|  | 15,920 | 5,413 | 8,918 | 0.7463 | 0.6410 | 0.6896 | 0.6046 | 0.2019 | 0.3 | 0.0 | 918 |
|  | 15,921 | 5,428 | 8,917 | 0.7457 | 0.6410 | 0.6894 | 0.6045 | 0.2019 | 0.3 | 0.1 | 937 |
|  | 15,931 | 5,450 | 8,907 | 0.7451 | 0.6414 | 0.6894 | 0.6047 | 0.2019 | 0.3 | 0.2 | 957 |
|  | 15,933 | 5,489 | 8,905 | 0.7438 | 0.6415 | 0.6888 | 0.6030 | 0.2019 | 0.3 | 0.3 | 1,033 |
| YOLOv5s | 14,655 | 2,265 | 10,183 | 0.8661 | 0.5900 | 0.7019 | 0.5792 | 0.2019 | 0.3 | - | 164 |

## **Table S6.** Class-agnostic arthropod localisation performance metrics for the optimised YOLOv5-small model (YOLOv5s) alone and for the combined results of YOLOv5s with SAHI enhancement applied to images where the YOLOv5s model did not detect arthropods (YOLOv5s + SAHI). The SAHI hyperparameters were: inference slice size (640 x 640 pixels to match training image size), confidence (Conf.), intersection over union (IoU), and slice overlap ratio (Overlap). Metrics include: true positives (TP), false positives (FP), false negatives (FN), precision (P), recall (R), harmonic mean of P and R (F1), and area under the P-R curve (AUC). The SAHI results are sorted by F1 in descending order.


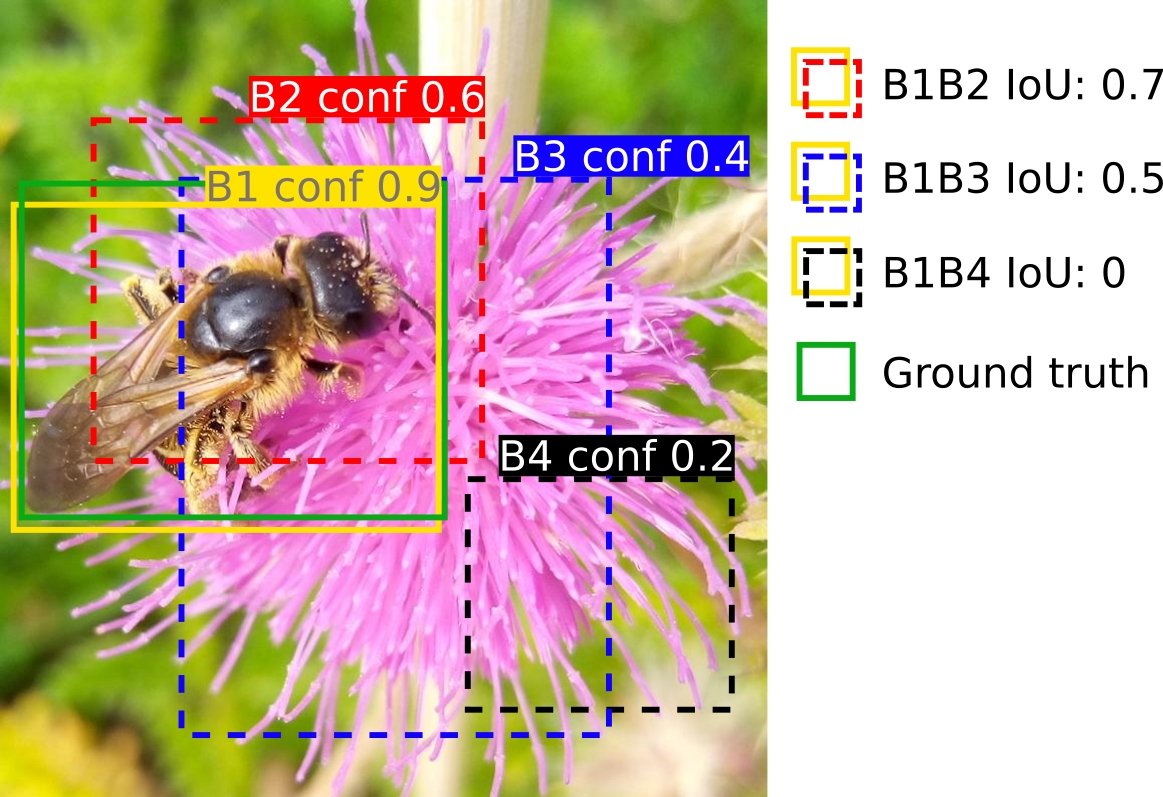


## **Figure S1.** Illustration of Non-Maximum Suppression (NMS) with a confidence (NMS-conf) threshold of 0.3 and an Intersection-over-Union (NMS-IoU) threshold of 0.4. NMS operates independently of the ground truth (GT) box, processing only predicted bounding boxes (B1-B4). First, predicted boxes with confidence below the NMS-conf of 0.3 are discarded (e.g., B4 conf 0.2) irrespective of whether they overlap or not. The highest-confidence box (e.g., B1 conf 0.9) is selected, and boxes with IoU ≥ NMS-IoU 0.4 relative to B1 are suppressed (e.g., B1-B2 IoU: 0.7, B1-B3 IoU: 0.5), removing redundant predicted boxes for the same GT box. This process would iterate with the next highest-confidence box among any remaining predictions that were not discarded by the IoU ≥ NMS-IoU filter. For example, at an NMS-IoU of 0.6, B1 and B3 are retained, while only B2 is discarded. Next, after the NMS algorithm, if the GT overlaps well with the predicted box B1 (IoU ≥ eval-IoU 0.5), B1 is classified as a true positive (TP) and B3 as a false positive (FP). Note that B1 could be marked as a FP if it overlapped insufficiently with the GT, and the GT would then be marked as a false negative (FN). So, a higher NMS-IoU threshold permits overlapping predictions, aiding localisation of closely spaced arthropods, though it may increase false positives (FPs). Conversely, a lower NMS-IoU threshold is more aggressive in discarding predictions and is better suited for solitary arthropods. Operating after the NMS, the eval-IoU threshold impacts performance metrics by defining TPs, FPs and FNs. The IoU overlaps of the predicted bounding boxes are approximate and intended for illustration purposes only.


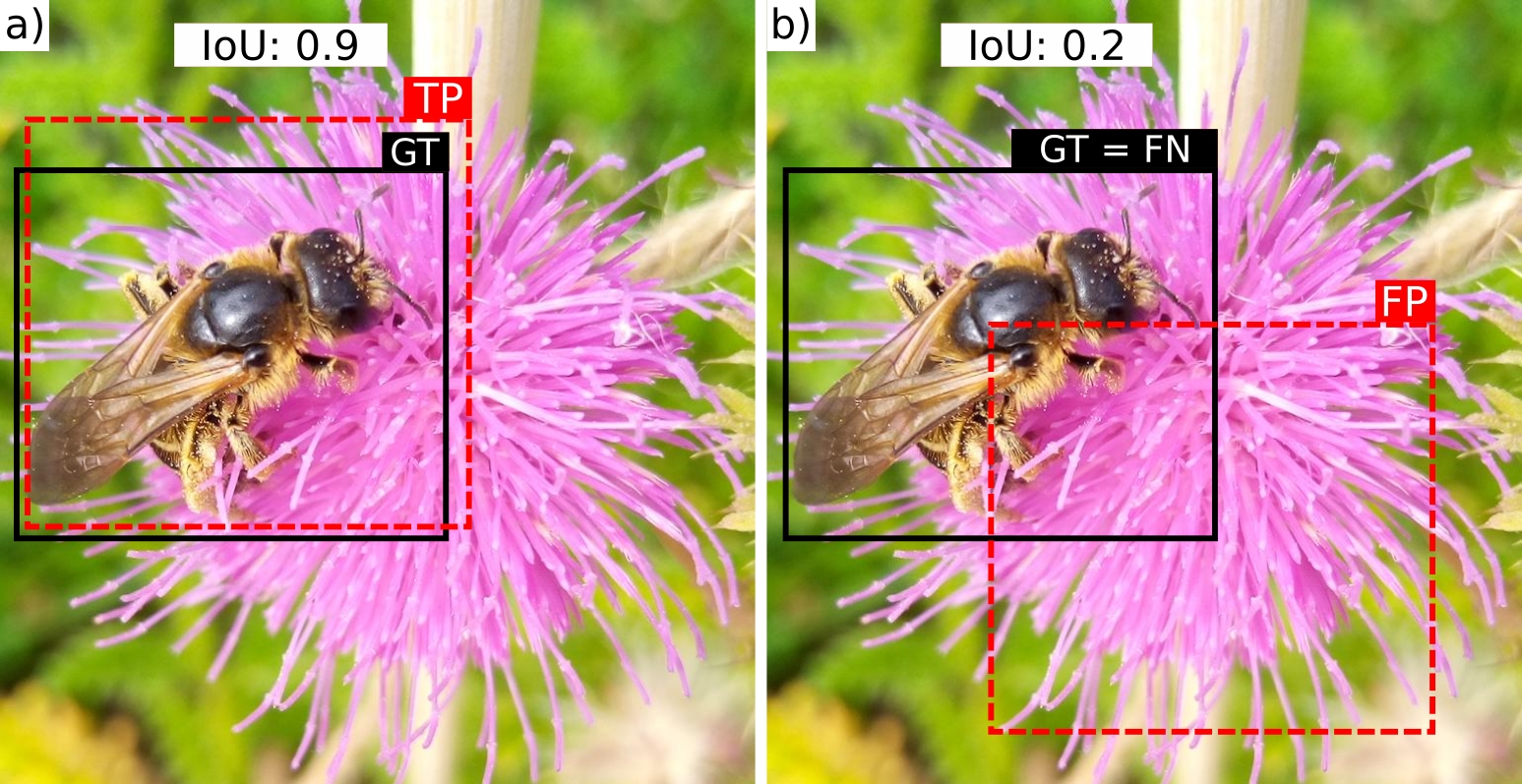


## **Figure S2.** Illustration of the concepts of true positive (box-TP), false positive (box-FP), and false negative (box-FN) using an eval-IoU threshold of 0.5. True negatives (TN) are not applicable in this object localisation context. Panel a) shows an IoU of 0.9 between the predicted box and the ground truth (GT), classifying it as a TP. Panel b) shows an IoU of 0.2, resulting in the prediction being labelled as a FP and the GT as a FN. The IoU overlaps of the bounding boxes are approximate and intended for illustration purposes only.
